# Separation of fission produced ^106^Ru from simulated high level nuclear wastes for production of brachytherapy sources

**DOI:** 10.1007/s10967-013-2570-3

**Published:** 2013-06-20

**Authors:** Magdalena Blicharska, Barbara Bartoś, Seweryn Krajewski, Aleksander Bilewicz

**Affiliations:** Institute of Nuclear Chemistry and Technology, Dorodna 16, 03-195 Warsaw, Poland

**Keywords:** ^106^Ru, Brachytherapy, Separation techniques

## Abstract

An effective and simple process for the isolation of ^106^Ru from high-level liquid wastes was developed. Radioactive ruthenium was oxidized by H_5_IO_6_ in HNO_3_ solution and was extracted to CCl_4_ phase in the form of RuO_4_. In order to obtain ruthenium in the suitable form for production of brachytherapy sources, RuO_4_ in organic phase was reduced and re-extracted to aqueous phase. The efficiency of extraction of ^103^Ru to organic phase was 86 %, re-extraction to aqueous solution was near 100 %, so the overall recovery of ^103^Ru is estimated at more than 80 %.

## Introduction

Brachytherapy is the common method for treating various tumors, and currently the ruthenium-106 and iodine-125 applicators are the most frequently used. Considering that ^106^Ru is a *β* ^*−*^ emitter with maximum energy of 3.54 MeV, it is best indicated in the treatment of small melanomas, with up to 20 mm tissue range [1]. It also replaced ^90^Sr/^90^Y sources because of it higher energy of emitted *β* ^*−*^ particles [2] and possibility of simpler source preparation.

^106^Ru is commercially obtained from neutron irradiated high enrichment ^235^U target in process of production ^99^Mo. After isolation of ^99^Mo radioisotope and decaying of ^103^Ru, ruthenium is separated from the wastes by multistep procedure. At present, there are only a handful of ageing reactors worldwide capable of producing the ^99^Mo, therefore alternative strategies for production of this key medical isotope are explored. In our work, we propose to use liquid high-level radioactive waste as a source of high activity of ^106^Ru.

The potential utilisation of fission-produced platinum metals (fission platinoids) as valuable products has attracted attention in the last few decades, as large amounts of spent nuclear fuel have accumulated worldwide [3]. Table 1 presents the isotopic composition of ruthenium isotopes after 5 years cooling of liquid nuclear waste [4].

**Table 1:** Isotopic composition of ruthenium originated from fission of ^235^U after 5 years cooling

Isotope	Content weight (%)	Half-life	Specific activity (GBq g^−1^)
99	Trace	Stable	–
100	4.2	Stable	–
101	34.1	Stable	–
102	34.0	Stable	–
103	Ultra trace	39 days	–
104	23.9	Stable	–
106	3.8	1 year	300

Simple calculations indicate that 1 dm^3^ of waste solution after reprocessing of nuclear fuel contains about 500 GBq of ^106^Ru after 4 years of cooling. This amount of activity is enough for production of about few thousands of brachytherapy sources.

During reprocessing of the spent fuel, the metallic Ru is dissolved in concentrated nitric acid and forms stable Ru-nitrosyl complexes [5]. In the high acidity the dominating ruthenium species are the [RuNO(NO_2_)_2_(NO_3_)(H_2_O)_2_]^0^ and [RuNO(NO_2_)_2_(H_2_O)_3_]^+^ [6]. The concentration of different species depends mainly on the composition of the medium and also the time of ageing.

Ruthenium metal was efficiently separated from other fission products by oxidation and distillation of RuO_4_ with absorption in NaOH solution. El-Absy et al. [7, 8] separated Ru radionuclides from a ^131^I-free fission product acidic solution containing KMnO4, by boiling for 40 min. In other work, ruthenium was electrochemically eliminated from a 3 M HNO_3_ solution of high-level waste, as RuO_4_, in the presence of AgNO_3_ at 60 °C [9]. Gandon et al. [10] co-precipitated ruthenium with copper ferrocyanide neutral solution. D. Banerjee et al. [11] used conventional ion exchangers and chemical precipitation based processes for the effective removal of the ^106^Ru activity from NH_4_NO_3_ effluent generated during wet processing of rejected sintered depleted uranium fuel pellets.

Present communication reports results of our process development studies on the recovery of ruthenium radioisotopes from simulated solution of high level radioactive waste using oxidation-extraction method.

## Experimental

### Radionuclide

For reasons of availability we used in experiments the ^103^Ru nuclide instead of ^106^Ru. The latter nuclide ^106^Ru is separated in complicated procedure from fission products of ^235^U, while ^103^Ru is produced in a simple way by direct thermal neutron irradiation of natural ruthenium. ^103^Ru was obtained by neutron irradiation of ruthenium salt (NH_4_)_2_[RuCl_5_(H_2_O)] at a neutron flux 7 × 10^13^ n cm^−2^ s^−1^ for 8 h in the nuclear reactor Maria at Świerk, Poland. The irradiated target was dissolved in 1 M HNO_3_.

Others radionuclides, ^131^I in the form of Na^131^I solutions was obtained from NCBJ-Polatom Świerk and ^99m^Tc in the form of ^99m^TcO_4_^−^ was milked from ^99^Mo/^99m^Tc generator.

### Radioactivity measurements

The ^103^Ru radioactivity was measured using an ORTEC system with a high resolution HPGe detector using photo peak at *E*_γ_ = 497.05 keV (88.7 %) and in NaI γ-scintilation counter LG-1b, ICHTJ, Poland.

### Reagents

The following commercial chemicals were used without additional purification: H_5_IO_6_ was from Fluka and (NH_4_)_2_[RuCl_5_(H_2_O)] from Alfa Aesar, other reagents, carbon tetrachloride from Chempur, Poland, reductants and acids were from POCh Gliwice, Poland. Desirable concentrations of reagents were obtained by dilution of stock solutions.

### Solvent extraction studies

Experiments were carried out under ambient conditions by shaking equal volume (5 ml each) of organic and aqueous phase in a separatory funnel using wrist action shaker. Phase separation was done by centrifugation and suitable aliquots (1 ml) of each phase were assayed. The distribution ratio “D” of the metal was determined as the ratio of metal concentration in organic phase to that in aqueous phase. Percentage extraction of metal ion was calculated by equation:

$$ {\text{\% }}E\; = \;\frac{\text{D}}{{{\text{D}} + 1}}\; \times \;100\;{\text{\% }}. $$

## Results

### Extraction of ^103^Ru to CCl_4_ phase

In oxidizing solutions ruthenium forms tetroxide, RuO_4_, which is easily extractable to organic phase. Formation of RuO_4_ is indicated by color change from deep orange to golden yellow. The RuO_4_ formed was extracted to an organic phase. Unfortunately, the RuO_4_ is not stable in the CCl_4_ phase and formation of black RuO_2_ precipitate is observed after a few hours. To avoid reduction of RuO_4_ to RuO_2_ the organic phase was contacted with a solution generating Cl_2_ molecules: 0.01 M HCl + 0.05 M H_5_IO_6_. The Cl_2_ molecules, formed in aqueous solution, are very soluble in CCl_4_ and distributed among the two liquid phases keeping ruthenium in the form of RuO_4_ in the organic phase for several month [12].

Influence of the various oxidants and acids on ruthenium oxidation-extraction process were studied to optimize the process. Table 2 presents results of ^103^Ru extraction from solutions containing various oxidizing agents. Concentration of used oxidants was the same taking into account the number of electrons involved in the reaction.

**Table 2:** Efficiency of Ru extraction in various oxidizing solutions

Oxidant	Distribution coefficient	% of extraction
H_5_IO_6_	6.17	86.1
KIO_4_	3.02	75.1
KMnO_4_	3.88	79.6
K_2_Cr_2_O_7_	0.045	4.30

*Aqueous phase* 1 M HNO_3_, *organic phase* CCl_4_

As show in Table 2 the obtained results indicate that the best oxidant is orthoperiodic acid (86.0 % extraction), a somewhat worse, but also possible to use is a potassium metaperiodate (75.1 %) and potassium permanganate (79.5 %). The obtained results well correlate with oxidation potential of reagent used.

**Fig. 1:**
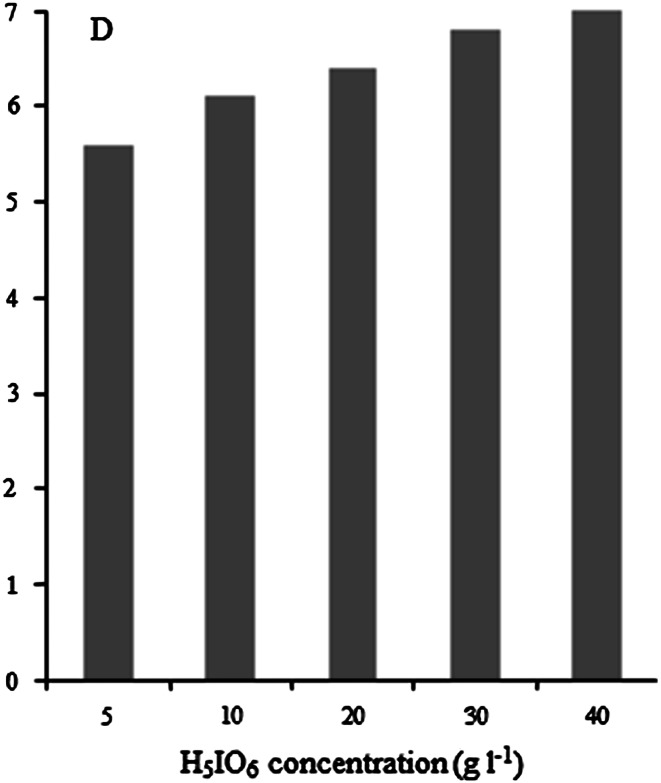
Extraction of ^103^Ru from 1 M HNO_3_ solution containing various concentration of H_5_IO_6_

An important parameter was the selection of a suitable amount of oxidant to obtain complete oxidation of ruthenium to RuO_4_ and thus its extraction into the organic phase. We have studied the ^103^Ru extraction depending on the concentration of orthoperiodic acid. The results are presented in Fig. 1.

In concentration range from 5 to 40 g l^−1^ of H_5_IO_6_ only insignificant increasing of ^103^Ru extraction is observed. Therefore, it can be assumed that that solution containing only 10 g l^−1^ of H_5_IO_6_ should be sufficient for effective extraction of ^103^Ru to CCl_4_ phase.

In the next step, influence of various acids and acid concentrations on ^103^Ru extraction were studied. We examined the following acids: nitric acid, sulfuric acid, hydrochloric acid and perchloric acid. The results are presented in Tables 3 and 4.

**Table 3:** Efficiency of Ru extraction in various acid solutions

Acid (1 M)	Distribution coefficient
HNO_3_	6.17
H_2_SO_4_	6.25
HClO_4_	6.34
HCl	4.21

H_5_IO_6_—10 g l^−1^

**Table 4:** Extraction of ^103^Ru in various HNO_3_ concentrations, concentration of H_5_IO_6_—10 g l^−1^

HNO_3_ (M)	Distribution coefficient
1	6.17
2	6.26
5	6.71

In the solutions of HNO_3_, H_2_SO_4_, and HClO_4_ extraction of ^103^Ru was comparable. Only in HCl solution extraction was significantly lower. Additionally, the use of HCl solution is not desirable due to the formation of Cl_2_ gas by reaction of orthoperiodic with hydrochloric acid. For further experiments HNO_3_ solution was selected. This choice was dictated by the fact that the high-level radioactive waste are generally in the form of a HNO_3_ solution. Table 3 presents dependence of the ^103^Ru extraction on the HNO_3_ concentration in range of 1–5 M.

As shown in the Table 4 only very small increasing of ^103^Ru extraction was observed when HNO_3_ concentration increased from 1 to 5 M. Summarizing our results on optimization of ^103^Ru extraction process, we can conclude that 86 % of extraction could be obtained for using H_5_IO_6_—10 g l^−1^ as oxidant and 1 M HNO_3_ solution. Using of higher H_5_IO_6_ and HNO_3_ concentrations gave only insignificant increasing of the process efficiency.

The PUREX raffinate contains also other long-lived fission products like ^135,137^Cs, ^90^Sr, ^241^Am, ^99^Tc, ^129^I, ^97^Zr, among which ^99^Tc and ^129^I could be potentially co-extract with ^106^Ru. In oxidizing solution technetium could be extracted as HTcO_4_ and iodine in I_2_ or interhalogen form. The ^135,137^Cs, ^90^Sr, ^241^Am and other metallic radionuclides in HNO_3_ solution, not containing complexing agents, are present in either cationic form or nonextractable species.

For co-extraction studies of ^99^Tc and ^129^I we used short-lived isotopes ^99m^Tc and ^131^I. The extraction of both radionuclides were performed in solution of concentration of 10 g l^−1^ H_5_IO_6_ in 1 M HNO_3_. We did not observe extraction of radionuclide studied, radioactivity of the ^99m^Tc and ^131^I in the organic phase was below the background level.

Since the ^106^Ru sources for brachytherapy are usually obtained by electrochemical deposition from aqueous solutions [13], we investigated the possibility of ruthenium transfer from the organic to aqueous phase. Because RuO_4_ is the only form of ruthenium, which is stable in CCl_4_ phase, for re-extraction of ^103^Ru we decided to reduce RuO_4_ to Ru(III) and Ru(II) oxidation state. The following compounds were selected as reductants: sodium sulfite, hydroxylamine, hydrazine and sodium borohydride. Results of ^103^Ru extraction from the organic into aqueous phase are shown in Table 5.

**Table 5:** Percent of re-extraction of ^103^Ru from CCl_4_ phase to solution containing reducing agent

Reducing solution	% of re-extraction
0.1 M Na_2_SO_3_ + 0.01 M HCl	83.6
0.1 M Na_2_SO_3_ + 0.1 M HCl	89.3
0.1 M Na_2_SO_3_ + 1 M HCl	94.9
1 M Na_2_SO_3_ + 1 M HCl	96.1
0.1 M NH_2_OH	95.0
0.1 M NH_2_OH + 0.1 HCl	100
0.1 M N_2_H_4_	100
0.1 M N_2_H_4_ + 0.1 HCl	92.6
0.1 M NaBH_4_	87.0

As shown in Table 5, the best results were obtained for 0.1 M aqueous solutions of hydrazine and for hydroxylamine hydrochloride. These reductants are most sufficient, because of their relatively high solubility in the organic phase, where reduction of RuO_4_ to the Ru(III) and Ru(II) took place. Reduced forms of ruthenium are insoluble in CCl_4_ phase and passed immediately to the aqueous phase.

**Fig. 2:**
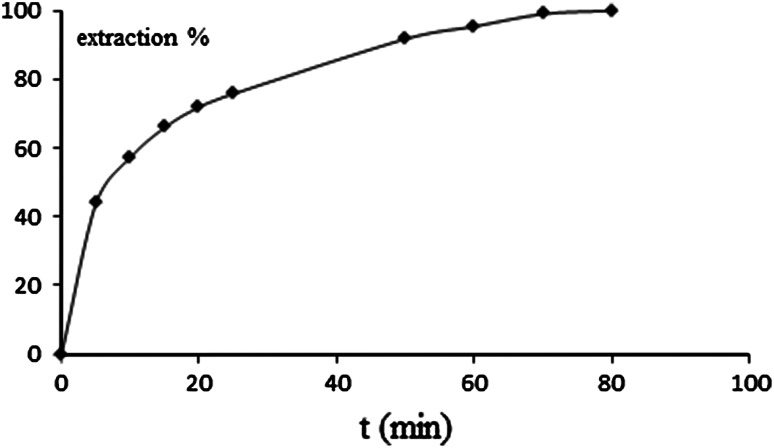
Kinetic of ^103^Ru re-extraction from CCl_4_ to aqueous phase (0.1 M hydrazine)

Kinetic studies were carried out in the system ^103^RuO_4_ in CCl_4_ (organic phase) and Na_2_SO_3_ 0.1 M HCl (aqueous phase). The results presented in Fig. 2 indicate that the process is relatively fast and after 40 min equilibrium state is achieved.

## Conclusion

A highly effective and flexible process for the separation of ^106^Ru from simulated high-level liquid waste was elaborated. It was found that the optimal way for extraction of ^103^Ru to CCl_4_ organic phase is oxidation of ruthenium nitrozyl complexes to RuO_4_ by 10 g l^−1^ H_5_IO_6_ in 1 M HNO_3_ solution. It was found that in re-extraction process to aqueous phase the most efficient compounds for reduction of RuO_4_ in CCl_4_ phase are hydrazine and hydroxylamine hydrochloride. The overall recovery of ^106^Ru is estimated at more than 80 %.

Production batches of hundreds GBq of ^106^Ru radioisotope separated from 1 l of PUREX raffinate can be achieved using the above-mentioned separation technique. For verification of the obtained results further experiments with real wastes solutions is necessary.
